# Outcomes among confirmed cases and a matched comparison group in the Long-COVID in Scotland study

**DOI:** 10.1038/s41467-022-33415-5

**Published:** 2022-10-12

**Authors:** Claire E. Hastie, David J. Lowe, Andrew McAuley, Andrew J. Winter, Nicholas L. Mills, Corri Black, Janet T. Scott, Catherine A. O’Donnell, David N. Blane, Susan Browne, Tracy R. Ibbotson, Jill P. Pell

**Affiliations:** 1 Institute of Health and Wellbeing, University of Glasgow, Glasgow, G12 8RZ UK; 2 Emergency Department, Queen Elizabeth University Hospital, Glasgow, G52 4TF UK; 3 Public Health Scotland, Meridian Court, Glasgow, G2 6QQ UK; 4 School of Health and Life Sciences, Glasgow Caledonian University, Glasgow, G4 0BA UK; 5 Sandyford Sexual Health Services, NHS Greater Glasgow and Clyde, Glasgow, G3 7NB UK; 6 BHF Centre for Cardiovascular Science, University of Edinburgh, Edinburgh, EH16 4SU UK; 7 Usher Institute, University of Edinburgh, Edinburgh, EH16 4UX UK; 8 Aberdeen Centre for Health Data Science, University of Aberdeen, Aberdeen, AB25 2ZD UK; 9 Public Health Directorate, NHS Grampian, Aberdeen, AB15 6RE UK; 10 MRC-University of Glasgow Centre for Virus Research, University of Glasgow, Glasgow, G61 1QH UK

**Keywords:** Epidemiology, Risk factors, SARS-CoV-2

## Abstract

With increasing numbers infected by SARS-CoV-2, understanding long-COVID is essential to inform health and social care support. A Scottish population cohort of 33,281 laboratory-confirmed SARS-CoV-2 infections and 62,957 never-infected individuals were followed-up via 6, 12 and 18-month questionnaires and linkage to hospitalization and death records. Of the 31,486 symptomatic infections,1,856 (6%) had not recovered and 13,350 (42%) only partially. No recovery was associated with hospitalized infection, age, female sex, deprivation, respiratory disease, depression and multimorbidity. Previous symptomatic infection was associated with poorer quality of life, impairment across all daily activities and 24 persistent symptoms including breathlessness (OR 3.43, 95% CI 3.29–3.58), palpitations (OR 2.51, OR 2.36–2.66), chest pain (OR 2.09, 95% CI 1.96–2.23), and confusion (OR 2.92, 95% CI 2.78–3.07). Asymptomatic infection was not associated with adverse outcomes. Vaccination was associated with reduced risk of seven symptoms. Here we describe the nature of long-COVID and the factors associated with it.

## Abstract (web summary)

In this population-based cohort study from Scotland, the authors investigate the prevalence of symptoms in the post-acute phase of COVID-19 infection compared to matched uninfected controls. They identify persistent symptoms associated with infection and identify factors associated with failure to recover.

## Introduction

Whilst most people recover fully from severe acute respiratory syndrome coronavirus-2 (SARS-CoV-2) infection, some develop long-COVID. With increasing numbers of people having been infected since the start of the pandemic, attention is shifting from managing the acute infection to understanding long-COVID in order to inform the health and social care response. The WHO defined long-COVID as “a history of probable or confirmed SARS-CoV-2 infection … with symptoms that last for at least 2 months and cannot be explained by an alternative diagnosis”^1^. The imprecision of this, and other, definitions reflects our poor understanding of the nature of long-COVID and its underlying mechanisms.

A meta-analysis of 63 studies pooled symptom prevalence following laboratory-confirmed SARS-CoV-2 infection^2^. The sample size ranged from 58 to 1733 (16,336 in total), with most studies containing fewer than 200 participants. Overall, 53% of people reported one or more symptoms beyond 12 weeks following infection. The most common were fatigue, pain/discomfort, shortness of breath, cognitive impairment, and mental health problems. Eighteen studies were judged to be at high risk of bias, and the remaining 45 were at moderate risk, due to convenience sampling and unrepresentative study populations. Half of the studies investigated hospitalised cohorts. The only two studies with more than 1000 participants were a hospitalised cohort and a cohort of health-care workers with mild infection. In a subsequent meta-analysis of 18 studies with at least 12 months follow-up, study samples ranged from 51 to 2433 (8591 in total) and 28% of participants reported fatigue/weakness, 22% anxiety, 18% breathlessness, 19% memory loss, 18% concentration difficulties and 12% insomnia^3^. The authors highlighted a small sample size, lack of representativeness and low response rate as limitations. Whilst many long-COVID studies have focused on patients hospitalised with more severe infections, a meta-analysis of 9 studies reported persistent symptoms in up to one-third of people following mild SARS-CoV-2 infection^4^.

This study aimed to address some of the limitations of existing studies by determining the frequency, nature, determinants and impact of long-COVID in the general population using a largescale, nationwide study, including people who had severe, mild and asymptomatic infections and a never infected comparison group, and measuring serial self-reported outcomes as well as outcomes obtained from linkage to routine health records. Here we show that the long-term sequelae of COVID-19 are wide-ranging and impact on all aspects of daily living, are specific to symptomatic infections and more likely following severe infections requiring hospitalisation, and in older, female and more deprived patients with pre-existing health problems but vaccination appears to confer some protection.

## Results

### Cohort characteristics

Overall, 102,473 (16%) of the 638,125 people invited consented and completed at least one questionnaire: 33,281 (20%) of the 162,957 people who had a positive test, and 69,192 (15%) of the 475,168 invited following only negative tests. Completion rates were 14% (90,578/625,315), 9% (21,963/242,412) and 11% (934/8625) for the 6, 12 and 18 month follow-up questionnaires respectively (Supplementary Table 1). Compared with those who did not provide consent, responders were more likely to be female (60.9% vs 51.2%; *p*-value < 0.001), were older (>40 years 59.5% vs 46.0%; *p*-value < 0.001) and less deprived (most deprived SIMD quintile 24.0% vs 27.0%; *p*-value <0.001).

We excluded 6235 participants who had only negative tests recorded but self-reported they had tested positive. Therefore, the study cohort comprised 96,238 participants. Their median age at baseline was 45 (IQR 31–56) years, 39% were male, 91% white, 30% had at least one pre-existing health condition and 16% at least two; 4% had received at least one COVID-19 vaccination dose prior to their index test (Table 1).

**Table 1:** Characteristics of study participants by infection status and symptomatology

	Never infected *N* = 62,957	Asymptomatic infection *N* = 1,795	Symptomatic infection *N* = 31,486	*P* value* Never versus asymptomatic	*P* value* Never versus symptomatic	Overall *N* = 96,238
	Med (IQR)	Med (IQR)	Med (IQR)			Med (IQR)
Age (years)	45 (31–56)	43 (28–57)	44 (30–56)	<0.001	<0.001	45 (31–56)
**Sex**	*N* (%)	*N* (%)	*N* (%)			*N* (%)
Female	37,422 (59.44)	957 (53.31)	20,202 (64.16)	<0.001	<0.001	58,581 (60.87)
Male	25,535 (40.56)	838 (46.69)	11,284 (35.84)			37,657 (39.13)
**SIMD**						
1 (most deprived)	14,906 (23.68)	504 (28.08)	7658 (24.32)	<0.001	0.044	23,068 (23.97)
2	13,594 (21.59)	418 (23.29)	6782 (21.54)			20,794 (21.61)
3	11,344 (18.02)	291 (16.21)	5656 (17.96)			17,291 (17.97)
4	11,632 (18.48)	289 (16.10)	5717 (18.16)			17,638 (18.33)
5 (least deprived)	11,481 (18.24)	293 (16.32)	5673 (18.02)			17,447 (18.13)
**Ethnic group**						
White	56,729 (90.11)	1207 (67.24)	29,286 (93.01)	<0.001	<0.001	87,222 (90.63)
South Asian	881 (1.40)	44 (2.45)	724 (2.30)			1649 (1.71)
Black	322 (0.51)	15 (0.84)	185 (0.59)			522 (0.54)
Other	1058 (1.68)	16 (0.89)	488 (1.55)			1562 (1.62)
Missing	3967 (6.30)	513 (28.58)	803 (2.55)			5283 (5.49)
**Number of pre-existing health conditions**						
0	43,008 (68.31)	1512 (84.23)	22,764 (72.30)	<0.001	<0.001	67,284 (70)
1	8616 (13.69)	152 (8.47)	4052 (12.87)			12,820 (13)
2–3	8975 (14.26)	111 (6.18)	3801 (12.07)			12,887 (13)
≥4	2358 (3.75)	20 (1.11)	869 (2.76)			3247 (3.4)
**Pre-existing health conditions**						
Arthritis	4063 (6.45)	56 (3.12)	1759 (5.59)	<0.001	<0.001	5878 (6.11)
Asthma/bronchitis/COPD	15,128 (24.03)	356 (19.83)	6,715 (21.33)	<0.001	<0.001	22,199 (23.07)
Cancer	969 (1.54)	13 (0.72)	304 (0.97)	0.005	<0.001	1286 (1.34)
CHD	2144 (3.41)	32 (1.78)	882 (2.80)	<0.001	<0.001	3058 (3.18)
Cystic fibrosis	21 (0.03)	≤10	≤10	0.439	0.127	≤10
Deep vein thrombosis	221 (0.35)	≤10	83 (0.26)	0.608	0.025	≤10
Depression/anxiety	29,996 (47.65)	657 (36.60)	13,501 (42.88)	<0.001	<0.001	44,154 (45.88)
Diabetes	3016 (4.79)	64 (3.57)	1380 (4.38)	0.016	0.005	4460 (4.63)
High blood pressure	5499 (8.73)	80 (4.46)	2,31 (8.36)	<0.001	0.051	8210 (8.53)
HIV	73 (0.12)	≤10	33 (0.10)	0.195	0.630	≤10
Home oxygen	34 (0.05)	≤10	16 (0.05)	0.325	0.841	≤10
Kidney disease	426 (0.68)	≤10	166 (0.53)	0.009	0.006	≤10
Liver disease	299 (0.47)	≤10	89 (0.28)	0.026	<0.001	≤10
Neurological condition	1565 (2.49)	17 (0.95)	505 (1.60)	<0.001	<0.001	2087 (2.17)
Overweight	5832 (9.26)	56 (3.12)	2568 (8.16)	<0.001	<0.001	8456 (8.79)
Obese	2302 (3.66)	20 (1.11)	928 (2.95)	<0.001	<0.001	3250 (3.38)
Pulmonary embolism	227 (0.36)	≤10	87 (0.28)	0.011	0.034	≤10
Pulmonary fibrosis	52 (0.08)	≤10	14 (0.04)	0.223	0.037	≤10
Stroke	536 (0.85)	≤10	198 (0.63)	0.035	<0.001	≤10
**Number of vaccinations**						
0	60,230 (95.67)	1687 (93.98)	30,440 (96.68)	0.002	<0.001	92,357 (95.97)
1	2361 (3.75)	95 (5.29)	979 (3.11)			3435 (3.57)
2–4	366 (0.58)	13 (0.72)	67 (0.21)			446 (0.46)
**Variant period**						
Pre VOC	28,730 (45.63)	803 (44.74)	15,213 (48.32)	0.017	<0.001	44,746 (46.50)
No dominant (1)	25,681 (40.79)	705 (39.28)	12,448 (39.54)			38,834 (40.35)
Alpha	6863 (10.90)	238 (13.26)	3064 (9.73)			10,165 (10.56)
No dominant (2)	1683 (2.67)	49 (2.73)	761 (2.42)			2493 (2.59)

*N* number, *Med* median, *IQR* inter-quartile range, *SIMD* Scottish Index of Multiple Deprivation, *COPD* chronic obstructive pulmonary disease, *CHD* coronary heart disease, *VOC* variant of concern.

*Mann–Whitney U test for age; *χ*^2^ test for nominal categorical variables; *χ*^2^ test for trend for ordinal categorical variables. All tests are two-sided.

### Symptoms during acute infection

Among the 33,281 participants who had a positive test, 31,486 (95%) were symptomatic at the time of infection; 1208 (4%) had one symptom, 1999 (6%) had two, 2493 (8%) had three, and 25,786 (82%) had more than three. Overall, 83% reported fatigue at the time of acute infection, 64% headache, 63% change in taste, 63% myalgia, 60% change in smell, 54% cough, 52% fever, 45% breathlessness, 41% loss of appetite, 38% joint pain, 31% sore throat, 23% diarrhea, 21% chest pain, 20% runny nose, 15% abdominal pain, 13% confusion, 13% hoarse voice, 9% hair loss, 8% ear pain, 2% reduced consciousness, and 0.3% seizures. Of those who reported symptom duration, 7259 (23%) reported <1 week, 13,710 (44%) 1–4 weeks, and 10,489 (33%) >4 weeks.

### Outcomes

Of the 31,486 people who had had symptomatic infections, 1856 (6%) reported that they had not recovered at all on their most recent follow-up questionnaire, and 13,350 (42%) that they had only partially recovered. Among the 1342 people whose infection required hospitalization, the figures were 217 (16%) and 797 (59%), respectively, and among the 30,096 managed in the community, they were 1639 (5%) and 12,553 (42%), respectively.

For the 3941 with serial questionnaire data there was little change in the overall breakdown; at their first follow-up, 316 (8%) had not at all recovered and 1866 (47%) had only partially recovered, compared to 324 (8%) and 1806 (46%), respectively, at their most recent follow-up. However, there was some cross-over between groups; 1453 (37%) remained fully recovered, 1372 (35%) remained partially recovered, and 175 (4%) continued to report no recovery, while 494 (13%) reported delayed recovery (improvement over time), and 447 (11%) reported relapse (deterioration over time).

The results were similar when specific follow-up periods were compared. In the sub-group of 3744 participants who had symptomatic infections and who completed questionnaires at both 6 and 12 months follow-up, the breakdown by no, partial and full recovery was 295 (8%) 1766 (47%), and 1683 (45%) at six months and 303 (8%), 1705 (46%) and 1736 (46%) at 12 months (Supplementary Table 2). Similarly, in the 197 participants who completed questionnaires at both 12 and 18 months follow-up, the figures were 21 (11%), 100 (51%) and 76 (39%), and 21 (11%), 101 (51%) and 75 (38%) at 12 and 18 months, respectively.

Of the 21,525 people with ongoing symptoms following symptomatic infection, the most common were tiredness, headache and muscle aches/weakness (Table 2). However, symptoms were also common among people never infected. Compared with the latter, people who had previous symptomatic infection were significantly more likely to report 24 of the 26 symptoms at follow-up after adjusting for potential confounders (Table 3). After changes in smell and taste, the largest effect sizes were observed for symptoms that were potentially cardiovascular in origin (breathlessness, chest pain and palpitations) and confusion (Table 3). People with previous symptomatic infection were also more likely to have multiple (≥3) symptoms than people never infected (14,236 (45%) vs 19,613 (31%)). There was weak evidence of clustering of musculoskeletal and neuropsychological symptoms following previous symptomatic infection (Supplementary Fig. 2). Among the participants who completed serial questionnaires, there was evidence of improvements in taste and smell between 6 and 12 months follow-up but increased reporting of a dry or productive cough between 6 and 18 months (Supplementary Table 3).

**Table 2:** Crude outcomes of participants by infection status and symptomatology

	Never infected *N* = 62,957	Asymptomatic infection *N* = 1795	Symptomatic infection *N* = 31,486	*P* value* Never versus asymptomatic	*P* value* Never versus symptomatic	Overall *N* = 96,238
**Symptoms in last week**	*N* (%)	*N* (%)	*N* (%)			*N* (%)
Tiredness	19,873 (31.57)	147 (8.19)	13,891 (44.12)	<0.001	<0.001	33,911 (35.24)
Headache	13,659 (21.70)	97 (5.40)	7658 (24.32)	<0.001	<0.001	21,414 (22.25)
Muscle aches/weakness	10,118 (16.07)	75 (4.18)	7559 (24.01)	<0.001	<0.001	17,752 (18.45)
Joint pain	8821 (14.01)	69 (3.84)	5865 (18.63)	<0.001	<0.001	14,755 (15.33)
Breathless	5078 (8.07)	50 (2.79)	6327 (20.09)	<0.001	<0.001	11,455 (11.90)
Anxious/depressed	9056 (14.38)	69 (3.84)	6180 (19.63)	<0.001	<0.001	15,305 (15.90)
Confused/difficulty concentrating	3187 (5.06)	23 (1.28)	3927 (12.47)	<0.001	<0.001	7137 (7.42)
Sleep problems	10,842 (17.22)	90 (5.01)	7487 (23.78)	<0.001	<0.001	18,419 (19.14)
Dry cough	4820 (7.66)	47 (2.62)	3556 (11.29)	<0.001	<0.001	8423 (8.75)
Cough with phlegm	5945 (9.44)	68 (3.79)	3198 (10.16)	<0.001	<0.001	9211 (9.57)
Change in taste	1048 (1.66)	11 (0.61)	2730 (8.67)	0.001	<0.001	3789 (3.94)
Change in smell	816 (1.30)	14 (0.78)	3278 (10.41)	0.055	<0.001	4108 (4.27)
Problems hearing	2024 (3.21)	16 (0.89)	1614 (5.13)	<0.001	<0.001	3654 (3.80)
Problems with eyesight	2300 (3.65)	17 (0.95)	2048 (6.50)	<0.001	<0.001	4365 (4.54)
Pins and needles	4043 (6.42)	33 (1.84)	3265 (10.37)	<0.001	<0.001	7341 (7.63)
Chest pain	2125 (3.38)	25 (1.39)	2017 (6.41)	<0.001	<0.001	4167 (4.33)
Palpitations	2355 (3.74)	19 (1.06)	2660 (8.45)	<0.001	<0.001	5034 (5.23)
Poor appetite	3145 (5.00)	18 (1.00)	1682 (5.34)	<0.001	0.023	4845 (5.03)
Abdominal pain	4161 (6.61)	24 (1.34)	2105 (6.69)	<0.001	0.657	6290 (6.54)
Sickness/vomiting	3713 (5.90)	16 (0.89)	1858 (5.90)	<0.001	0.984	5587 (5.81)
Diarrhea	4718 (7.49)	24 (1.34)	2468 (7.84)	<0.001	0.060	7210 (7.49)
Constipation	2444 (3.88)	19 (1.06)	1416 (4.50)	<0.001	<0.001	3879 (4.03)
Weight loss	821 (1.30)	≤10	629 (2.00)	0.003	<0.001	≤10
Dizzy/blackouts/fits	2033 (3.23)	18 (1.00)	1503 (4.77)	<0.001	<0.001	3554 (3.69)
Balance problems	2133 (3.39)	11 (0.61)	1799 (5.71)	<0.001	<0.001	3943 (4.10)
Skin rash	1726 (2.74)	19 (1.06)	1202 (3.82)	<0.001	<0.001	2947 (3.06)
No symptoms now	29,316 (46.57)	1428 (79.55)	9961 (31.64)	<0.001	<0.001	40,705 (42.30)
**Difficulties with activities of daily living**						
Walking; getting around	8359 (13.28)	98 (5.46)	5787 (18.38)	<0.001	<0.001	14,244 (14.80)
Housework; DIY; chores	8185 (13.00)	72 (4.01)	6178 (19.62)	<0.001	<0.001	14,435 (15.00)
Working; studying	7026 (11.16)	57 (3.18)	6181 (19.63)	<0.001	<0.001	13,264 (13.78)
Washing; dressing	3917 (6.22)	35 (1.95)	1829 (5.81)	<0.001	0.012	5781 (6.01)
Exercise; sport	8859 (14.07)	85 (4.74)	7939 (25.21)	<0.001	<0.001	16,883 (17.54)
Hobbies	4182 (6.64)	38 (2.12)	3,142 (9.98)	<0.001	<0.001	7362 (7.65)
Relationships	4735 (7.52)	51 (2.84)	3,191 (10.13)	<0.001	<0.001	7977 (8.29)
EQ-5D	*N* = 59,026	*N* = 1,289	*N* = 30,723			*N* = 91,038
Median (IQR)	80 (63–90)	90 (75–100)	75 (60–89)	<0.001	<0.001	78 (61–90)
Admitted to hospital	6409 (10.18)	141 (7.86)	3058 (9.71)	0.001	0.024	9608 (9.98)
Admitted to ICU	93 (0.15)	≤10	53 (0.17)	0.417	0.447	≤10
Died	72 (0.11)	≤10	21 (0.07)	0.517	0.028	≤10

*N* number, *DIY* do-it-yourself, *IQR* inter-quartile range, *ICU* intensive care unit.

*Mann–Whitney U test for EQ-5D, *χ*^2^ test for other variables. All tests are two-sided.

**Table 3:** Univariate and multivariate binary logistic regression analyses of the associations between previous symptomatic SARS-CoV-2 infection and current symptoms, referent to people never infected

	Univariate		Model 1*		Model 2**		Model 3***		Model 4 ****		Model 5 *****	
	OR (95% CI)	*p*-value	OR (95% CI)	*p*-value	OR (95% CI)	*p*-value	OR (95% CI)	*p*-value	OR (95% CI)	*p*-value	OR (95% CI)	*p*-value
**Sensory**												
Change in taste	5.61 (5.22, 6.03)	<0.001	5.47 (5.08, 5.88)	<0.001	5.68 (5.28, 6.12)	<0.001	5.70 (5.29, 6.13)	<0.001	5.68 (5.28, 6.11)	<0.001	5.69 (5.28, 6.12)	<0.001
Change in smell	8.85 (8.19, 9.57)	<0.001	8.62 (7.97, 9.32)	<0.001	8.79 (8.13, 9.51)	<0.001	8.79 (8.13, 9.51)	<0.001	8.78 (8.11, 9.49)	<0.001	8.77 (8.11, 9.49)	<0.001
Problems hearing	1.63 (1.52, 1.74)	<0.001	1.61 (1.50, 1.72)	<0.001	1.74 (1.63, 1.87)	<0.001	1.75 (1.63, 1.87)	<0.001	1.74 (1.63, 1.87)	<0.001	1.75 (1.63, 1.87)	<0.001
Problems with eyesight	1.83 (1.73, 1.95)	<0.001	1.82 (1.71, 1.94)	<0.001	1.98 (1.86, 2.11)	<0.001	1.99 (1.87, 2.12)	<0.001	1.98 (1.86, 2.11)	<0.001	1.99 (1.87, 2.11)	<0.001
Pins and needles	1.69 (1.61, 1.77)	<0.001	1.63 (1.55, 1.71)	<0.001	1.79 (1.71, 1.88)	<0.001	1.80 (1.71, 1.89)	<0.001	1.79 (1.71, 1.88)	<0.001	1.79 (1.71, 1.89)	<0.001
**Cardiorespiratory**												
Chest pain	1.96 (1.84, 2.09)	<0.001	1.90 (1.78, 2.02)	<0.001	2.09 (1.96, 2.22)	<0.001	2.10 (1.97, 2.23)	<0.001	2.09 (1.96, 2.22)	<0.001	2.09 (1.96, 2.23)	<0.001
Palpitations	2.37 (2.24, 2.51)	<0.001	2.27 (2.14, 2.40)	<0.001	2.50 (2.36, 2.66)	<0.001	2.51 (2.36, 2.66)	<0.001	2.51 (2.36, 2.66)	<0.001	2.51 (2.36, 2.66)	<0.001
Breathlessness	2.87 (2.75, 2.98)	<0.001	2.87 (2.75, 2.99)	<0.001	3.43 (3.28, 3.58)	<0.001	3.43 (3.29, 3.58)	<0.001	3.43 (3.28, 3.58)	<0.001	3.43 (3.29, 3.58)	<0.001
Dry cough	1.54 (1.47, 1.61)	<0.001	1.49 (1.42, 1.56)	<0.001	1.57 (1.50, 1.65)	<0.001	1.58 (1.51, 1.65)	<0.001	1.57 (1.50, 1.65)	<0.001	1.58 (1.51, 1.65)	<0.001
Cough with phlegm	1.08 (1.04, 1.13)	<0.001	1.06 (1.01, 1.10)	0.021	1.13 (1.08, 1.18)	<0.001	1.14 (1.08, 1.19)	<0.001	1.13 (1.08, 1.19)	<0.001	1.14 (1.08, 1.19)	<0.001
**Gastrointestinal**												
Poor appetite	1.07 (1.01, 1.14)	0.023	1.02 (0.96, 1.09)	0.472	1.13 (1.06, 1.20)	<0.001	1.13 (1.06, 1.21)	<0.001	1.13 (1.06, 1.20)	<0.001	1.13 (1.06, 1.20)	<0.001
Abdominal pain	1.01 (0.96, 1.07)	0.657	0.96 (0.91, 1.01)	0.111	1.04 (0.99, 1.10)	0.121	1.05 (0.99, 1.11)	0.102	1.05 (0.99, 1.11)	0.112	1.05 (0.99, 1.11)	0.101
Sickness/vomiting	1.00 (0.94, 1.06)	0.984	0.94 (0.89, 1.00)	0.055	1.04 (0.98, 1.10)	0.244	1.04 (0.98, 1.10)	0.215	1.04 (0.98, 1.10)	0.229	1.04 (0.98, 1.10)	0.215
Diarrhea	1.05 (0.99, 1.10)	0.060	1.01 (0.96, 1.06)	0.666	1.10 (1.04, 1.16)	<0.001	1.10 (1.04, 1.16)	<0.001	1.10 (1.04, 1.16)	<0.001	1.10 (1.04, 1.16)	<0.001
Constipation	1.17 (1.09, 1.25)	<0.001	1.12 (1.04, 1.19)	0.001	1.23 (1.15, 1.31)	<0.001	1.23 (1.15, 1.32)	<0.001	1.23 (1.15, 1.32)	<0.001	1.23 (1.15, 1.32)	<0.001
**Musculoskeletal**												
Muscle aches/weakness	1.65 (1.60, 1.71)	<0.001	1.62 (1.57, 1.68)	<0.001	1.79 (1.73, 1.85)	<0.001	1.79 (1.73, 1.85)	<0.001	1.79 (1.73, 1.85)	<0.001	1.79 (1.73, 1.85)	<0.001
Joint pain	1.40 (1.35, 1.46)	<0.001	1.40 (1.35, 1.45)	<0.001	1.60 (1.51, 1.63)	<0.001	1.57 (1.51, 1.63)	<0.001	1.57 (1.51, 1.63)	<0.001	1.57 (1.51, 1.63)	<0.001
**Neurological/mental health**												
Headache	1.16 (1.12, 1.20)	<0.001	1.09 (1.05, 1.12)	<0.001	1.16 (1.12, 1.20)	<0.001	1.16 (1.12, 1.20)	<0.001	1.16 (1.12, 1.20)	<0.001	1.16 (1.12, 1.20)	<0.001
Anxious/depressed	1.45 (1.40, 1.51)	<0.001	1.38 (1.33, 1.43)	<0.001	1.58 (1.52, 1.64)	<0.001	1.58 (1.52, 1.64)	<0.001	1.58 (1.52, 1.64)	<0.001	1.58 (1.52, 1.64)	<0.001
Confused/difficulty concentrating	2.67 (2.54, 2.81)	<0.001	2.59 (2.46, 2.72)	<0.001	2.92 (2.77, 3.07)	<0.001	2.92 (2.78, 3.07)	<0.001	2.92 (2.77, 3.07)	<0.001	2.92 (2.78, 3.07)	<0.001
Sleep problems	1.50 (1.45, 1.55)	<0.001	1.45 (1.40, 1.50)	<0.001	1.59 (1.53, 1.64)	<0.001	1.59 (1.54, 1.65)	<0.001	1.59 (1.53, 1.64)	<0.001	1.59 (1.54, 1.64)	<0.001
Dizzy/blackouts/fits	1.50 (1.40, 1.61)	<0.001	1.43 (1.33, 1.53)	<0.001	1.57 (1.47, 1.69)	<0.001	1.58 (1.47, 1.69)	<0.001	1.57 (1.47, 1.69)	<0.001	1.58 (1.47, 1.69)	<0.001
Balance problems	1.73 (1.62, 1.84)	<0.001	1.72 (1.61, 1.83)	<0.001	1.94 (1.81, 2.07)	<0.001	1.94 (1.81, 2.07)	<0.001	1.93 (1.81, 2.07)	<0.001	1.94 (1.81, 2.07)	<0.001
**Non-specific**												
Tiredness	1.71 (1.66, 1.76)	<0.001	1.64 (1.60, 1.69)	<0.001	1.80 (1.74, 1.85)	<0.001	1.80 (1.75, 1.85)	<0.001	1.80 (1.74, 1.85)	<0.001	1.80 (1.75, 1.85)	<0.001
Weight loss	1.54 (1.39, 1.71)	<0.001	1.51 (1.36, 1.67)	<0.001	1.63 (1.47, 1.81)	<0.001	1.64 (1.47, 1.82)	<0.001	1.63 (1.47, 1.82)	<0.001	1.64 (1.47, 1.82)	<0.001
Skin rash	1.41 (1.31, 1.52)	<0.001	1.36 (1.26, 1.47)	<0.001	1.44 (1.33, 1.55)	<0.001	1.44 (1.33, 1.55)	<0.001	1.43 (1.33, 1.55)	<0.001	1.43 (1.33, 1.55)	<0.001
No symptoms now	0.53 (0.52, 0.55)	<0.001	0.56 (0.54, 0.57)	<0.001	0.50 (0.49, 0.52)	<0.001	0.50 (0.49, 0.52)	<0.001	0.50 (0.49, 0.52)	<0.001	0.50 (0.49, 0.52)	<0.001

Model 1 - adjusted for age, sex, ethnicity, SIMD quintile and stage of follow-up; Model 2 - further adjusted for baseline comorbidity count and asthma/bronchitis/COPD, CHD, depression, and diabetes; Model 3 – model 2 further adjusted for variant period; Model 4 - model 2 further adjusted for vaccination status; Model 5 – model 2 further adjusted for variant period and vaccination status.

*OR* odds ratio, *CI* confidence interval. All tests are two-sided.

Routine data were available until January 2022, providing a median (IQR) of 7 (6–8) months follow-up. People who had previous symptomatic infection were not at significantly increased risk of all-cause hospitalization (fully adjusted OR 1.02, 95% CI 0.97–1.07, *p* = 0.386), ICU admission (fully adjusted OR 1.21, 95% CI 0.86–1.70, *p* = 0.267) or all-cause mortality (fully adjusted OR 0.64, 95% CI 0.39–1.05, *p* = 0.076). However, they had a median EQ-5D score of 75 (IQR 60–89) in their most recent follow-up questionnaire compared with 80 (IQR 63–90) for people never infected (*p* < 0.001). People who had previous symptomatic infection had significantly lower EQ-5D scores in both the univariate Poisson model (coefficient 0.96, 95% CI 0.96–0.96, *p* < 0.001) and in the fully adjusted model (coefficient 0.95, 95% C I 0.95–0.95, *p* < 0.001). Similarly, people who had had symptomatic infection were significantly more likely to report impaired mobility, housework/chores, working/studying, washing/dressing, exercise/sport, hobbies and relationships after adjusting for potential confounders (Table 4). Asymptomatic SARS-CoV-2 infection was not associated with increased risk of current symptoms, impaired daily activities, reduced quality of life, hospitalization, ICU admission or death.

**Table 4:** Univariate and multivariate binary logistic regression analyses of the associations between previous symptomatic SARS-CoV-2 infection and current difficulties in activities of daily living, referent to people never infected

	Univariate		Model 1*		Model 2**		Model 3***		Model 4 ****		Model 5 *****	
	OR (95% CI)	*p*-value	OR (95% CI)	*p*-value	OR (95% CI)	*p*-value	OR (95% CI)	*p*-value	OR (95% CI)	*p*-value	OR (95% CI)	*p*-value
Walking; getting around	1.47 (1.42, 1.53)	<0.001	1.46 (1.41, 1.52)	<0.001	1.80 (1.73, 1.88)	<0.001	1.80 (1.73, 1.88)	<0.001	1.80 (1.73, 1.88)	<0.001	1.80 (1.73, 1.88)	<0.001
Housework; DIY; chores	1.63 (1.58, 1.69)	<0.001	1.58 (1.52, 1.64)	<0.001	1.96 (1.89, 2.04)	<0.001	1.97 (1.89, 2.05)	<0.001	1.97 (1.89, 2.05)	<0.001	1.97 (1.89, 2.05)	<0.001
Working; studying	1.94 (1.87, 2.02)	<0.001	1.84 (1.77, 1.91)	<0.001	2.08 (2.00, 2.16)	<0.001	2.08 (2.00, 2.16)	<0.001	2.08 (2.00, 2.16)	<0.001	2.08 (2.00, 2.16)	<0.001
Washing; dressing	0.93 (0.88, 0.98)	0.012	0.88 (0.83, 0.93)	<0.001	1.06 (0.99, 1.13)	0.053	1.06 (1.00, 1.13)	0.046	1.06 (1.00, 1.13)	0.053	1.06 (1.00, 1.13)	0.049
Exercise; sport	2.06 (1.99, 2.13)	<0.001	2.00 (1.93, 2.07)	<0.001	2.33 (2.25, 2.42)	<0.001	2.34 (2.25, 2.42)	<0.001	2.33 (2.25, 2.42)	<0.001	2.34 (2.25, 2.42)	<0.001
Hobbies	1.56 (1.48, 1.64)	<0.001	1.50 (1.42, 1.57)	<0.001	1.75 (1.66, 1.84)	<0.001	1.75 (1.67, 1.84)	<0.001	1.75 (1.66, 1.84)	<0.001	1.75 (1.67, 1.84)	<0.001
Relationships	1.39 (1.32, 1.45)	<0.001	1.32 (1.25, 1.38)	<0.001	1.53 (1.46, 1.61)	<0.001	1.54 (1.46, 1.61)	<0.001	1.53 (1.46, 1.61)	<0.001	1.53 (1.46, 1.61)	<0.001

Model 1 - adjusted for age, sex, ethnicity, SIMD quintile and stage of follow-up; Model 2 - further adjusted for baseline comorbidity count and asthma/bronchitis/COPD, CHD, depression, and diabetes; Model 3 – model 2 further adjusted for variant period; Model 4 - model 2 further adjusted for vaccination status; Model 5 – model 2 further adjusted for variant period and vaccination status

OR odds ratio; CI confidence interval; DIY do-it-yourself. All tests are two-sided.

### Factors associated with outcomes

Following previous symptomatic infection, lack of complete recovery was associated with more severe (hospitalized) initial infection, older age, female sex, deprivation, white ethnicity, and pre-existing health conditions, including respiratory disease and depression (Table 5). Compared to unvaccinated people, people vaccinated prior to symptomatic infection were less likely to report persistent change in smell (OR 0.58, 0.45–0.76), change in taste (OR 0.61, 95% CI 0.46–0.79), problems hearing (OR 0.60, 95% CI 0.44–0.83), poor appetite (OR 0.72, 95% CI 0.53–0.99), balance problems (OR 0.71, 95% CI 0.53–0.95), confusion/difficulty concentrating (OR 0.72, 95% CI 0.58–0.89), and anxiety/depression (OR 0.76, 95% CI 0.64–0.92) at their latest follow-up after adjustment for potential confounders.

**Table 5:** Multivariate logistic regression analysis of the factors associated with no or partial recovery, referent to full recovery, among people with previous symptomatic SARS-CoV-2 infection

		No recovery (*n* = 1,856)	Partial recovery (*n* = 13,350)
		OR (95% CI)	OR (95% CI)
Age		1.01 (1.01, 1.02)	1.01 (1.00, 1.01)
Sex	Female	1	1
	Male	0.70 (0.63, 0.78)	0.67 (0.64, 0.70)
Ethnicity	White	1	1
	South Asian	0.56 (0.36, 0.85)	0.57 (0.48, 0.68)
	Black	0.61 (0.30, 1.23)	0.41 (0.29, 0.59)
	Other	0.37 (0.21, 0.68)	0.54 (0.44, 0.66)
	Missing	0.61 (0.41, 0.89)	0.79 (0.67, 0.92)
SIMD quintile	1 (most deprived)	1	1
	2	0.82 (0.71, 0.94)	0.95 (0.88, 1.02)
	3	0.63 (0.54, 0.74)	0.89 (0.83, 0.96)
	4	0.53 (0.45, 0.62)	0.78 (0.73, 0.84)
	5 (least deprived)	0.40 (0.34, 0.48)	0.67 (0.62, 0.72)
Latest follow-up time	6 months	1	1
	12 months	1.59 (1.38, 1.84)	1.11 (1.03, 1.19)
	18 months	2.00 (1.26, 3.18)	1.34 (1.04, 1.73)
Baseline morbidity count	0	1	1
	1	1.23 (1.05, 1.43)	1.16 (1.07, 1.25)
	2–3	1.52 (1.30, 1.79)	1.47 (1.35, 1.60)
	≥4	2.21 (1.66, 2.95)	1.90 (1.58, 2.28)
Asthma/bronchitis/ COPD	No	1	1
	Yes	1.31 (1.16, 1.48)	1.19 (1.12, 1.27)
CHD	No	1	1
	Yes	1.03 (0.79, 1.36)	0.95 (0.81, 1.11)
Depression/anxiety	No	1	1
	Yes	2.29 (2.06, 2.55)	1.66 (1.58, 1.75)
Diabetes	No	1	1
	Yes	0.76 (0.60, 0.98)	0.83 (0.73, 0.95)
Variant period	Pre-VOC	1	1
	No dominant (1)	1.10 (0.96, 1.25)	0.98 (0.93, 1.04)
	Alpha	1.11 (0.91, 1.35)	0.96 (0.87, 1.05)
	No dominant (2)	0.83 (0.54, 1.27)	0.98 (0.83, 1.16)
Vaccinated at test	No	1	1
	Yes	0.92 (0.68, 1.26)	0.90 (0.78, 1.04)
Infection severity	Not hospitalised	1	1
	Hospitalised	4.60 (3.77, 5.61)	2.66 (2.32, 3.05)

*N* number, *OR* odds ratio, *CI* confidence interval, *SIMD* Scottish Index of Multiple Deprivation, *COPD* chronic obstructive pulmonary disease, *CVD* cardiovascular disease, *VOC* variant of concern. All tests are two-sided.

## Discussion

Between 6 and 18 months following symptomatic SARS-CoV-2 infection, almost half of those infected reported no, or incomplete, recovery. Whilst recovery status remained constant over follow-up for most, 13% reported improvement over time and 11% deterioration. Symptomatic SARS-CoV-2 infection was associated with a wide range of persistent symptoms, impaired daily activities and reduced health-related quality of life, independent of sociodemographic factors and pre-existing health conditions. The strongest associations were observed for symptoms that were potentially cardiovascular in origin (breathlessness, chest pain and palpitations) and confusion. Lack of recovery was associated with more severe infection, older age, female gender, white ethnicity, deprivation, pre-existing respiratory disease and multimorbidity but pre-infection vaccination was associated with reduced risk of some persistent symptoms. We found no evidence of sequelae following asymptomatic infection.

Our finding of impaired daily activities is consistent with previous studies. In a meta-analysis of 12 studies covering 4828 participants previously infected by SARS-CoV-2, 35% had problems with mobility, 8% with personal care, 42% pain/discomfort, and 38% anxiety/depression^5^. Similarly, in a global social media survey of 1020 confirmed and 2742 suspected previous COVID-19 cases, 45% reported an ongoing impact on their ability to work^6^. In line with our findings, previous studies have reported that women, older and more deprived people, and those with pre-existing health problems were less likely to recover completely from COVID-19^7–9^.

Of eight studies that assessed the effectiveness of pre-infection vaccination on long-COVID^10^, six (two cohort, two case-control, two cross-sectional) reported fewer symptoms 1–6 months following infection among those fully vaccinated^11–16^, including fatigue, headache, muscle weakness/pain, breathlessness, dizziness, and change in smell^13,16^. Our findings differ from previous studies^12–14,16^ in suggesting possible protection against persistent symptoms from even partial vaccination. Three studies have suggested that, among unvaccinated people, post-infection vaccination may also reduce the risk or severity of long-COVID^15,17,18^, especially, if given early following infection^15^.

As a general population study, our findings provide a better indication of the overall risk and burden of long-COVID than hospitalised cohorts. The inclusion of asymptomatic infections enabled us to demonstrate that long-COVID is specific to people with symptomatic infections. Incomplete ascertainment of all cases of COVID-19 was inevitable due to the lack of PCR testing at the beginning of the pandemic, followed by a gradual increase in testing capacity and therefore changes in testing criteria. The risk of misclassification was reduced by using a composite definition of the previous infection, requiring both laboratory confirmation and self-report, and excluding people who reported a previous positive test that was not on the database. The never-infected group will include some people with asymptomatic infection or symptomatic infection prior to testing becoming available. The latter could potentially lead to under-estimation of the true magnitude of association between SARS-CoV-2 infection and ongoing health problems.

Our cohort included a large sample (*n* = 33,281) of people previously infected and the response rate of 16% overall and 20% among people who had symptomatic infection was consistent with previous studies that have used SMS text invitations as the sole method of recruitment. For example, in a study on the impact of COVID-19, an SMS text invitation was sent to 7911 patients attending a rheumatology outpatient clinic, of whom 21% responded and 20% completed the questionnaire^19^. In another study, SMS text messages were sent to 350 cases recorded on a COVID-19 contact tracing database. Of these 24% responded and 1% provided the requested information on their contacts^20^. In our study, recruitment may have been lower in some sub-groups. For example, the questionnaire was written in English and accessed via a web-based app and therefore may have been inaccessible to people without internet access or without English as their first language. Formal and informal carers were permitted to assist respondents in completing the questionnaire. It is possible that people with persistent health problems may have been more motivated to participate. Whilst reporting of current symptoms will not be subject to recall bias, a potential study limitation is that symptoms at the time of acute infections were recalled at follow-up and, therefore, were potentially subject to recall bias.

Symptoms are common in the general population. The three most common symptoms among people previously infected were also reported by 16–32% of people never infected. Therefore, the inclusion of an uninfected comparison group enabled us to demonstrate that the outcomes were not due to confounding. This is a major strength of our study compared with other population cohort studies^9,18^. We matched our uninfected comparison group 3:1 at the time of invitation; allowing for an anticipated lower response rate among uninfected individuals and attrition due to subsequent infection. Serial outcome measurements in a sub-group of 3941 respondents enabled us to investigate the trajectory of long-COVID over time. Very few people had been fully vaccinated pre-infection. However, sufficient had received a single dose to demonstrate some evidence of protection against persistent symptoms. The non-significant lower risk of death following symptomatic infection is likely to reflect the fact that our study recruited people who had survived at least six months following infection.

Our finding of higher rates of recovery among black and South Asian participants is consistent with a previous study that observed a lower probability of persistent symptoms among Asian participants^9^. Apparently better long-term prognosis contrasts with the known higher risk of black and South Asian individuals during acute infection with SARS-CoV-2^21^ and may reflect survival bias, since participants needed to have survived at least 6 months following their acute infection to participate in our study. The Scottish population is 96% white^22^. Therefore, it is important that ethnic-specific outcomes are reported by other long-COVID studies with more ethnically diverse populations.

In conclusion, 6–18 months following symptomatic SARS-CoV-2 infection, adults were at greater risk of a diverse group of symptoms, poorer quality of life and wide-ranging impairment of their daily activities, which could not be explained by confounding. Sequelae were more likely following severe infection and were not observed following asymptomatic infection and pre-infection vaccination may be protective.

## Methods

### Study design

Long-COVID in Scotland Study (Long-CISS) is an ambidirectional, general population cohort. Every adult (>16 years) in Scotland with a positive PCR test for SARS-CoV-2 from April 2020 was invited to participate along with a comparison group who had had a negative test but never had a positive test, matched 3:1 by age, sex, and area-based socioeconomic deprivation quintile. The National Health Service (NHS) Scotland platform that provides PCR result notifications identified eligible participants and invited them via automated SMS text messages. The study commenced in May 2021 and was recruited both retrospectively and prospectively based on existing and new test results, respectively. People in the comparison group were reallocated to the infected group if, and when, they had a positive test. The cohort included people with asymptomatic SARS-CoV-2 infection detected, for example, during occupational or travel-related screening. Participants provided electronic consent and study approval was obtained from the West of Scotland Research Ethics Committee (ref. 21/WS/0020) and the Public Benefit and Privacy Panel (ref. 2021-0180).

### Data sources

A self-completed online questionnaire (Supplementary Fig. 1) collected information on pre-existing health conditions at the time of the index test (first positive test or, for the comparison group, most recent negative test) as well as current symptoms, limitations in daily activities and quality of life. Those who had tested positive also provided information on symptoms during the initial infection and current recovery status. Questionnaires were completed 6, 12 and 18 months after the index test. Additional data were obtained through linkage to electronic health records both five years prior to their index test and subsequent to the test (up to January 2022) on hospitalizations (Scottish Morbidity Record 01/04), dispensed prescriptions (Prescribing Information System), vaccinations, and death certificates (General Registrar Office). Long-CISS is ongoing and the findings in this manuscript relate to index tests performed up to May 2021 and follow-up questionnaires up to November 2021.

### Definitions

Infection was defined as a positive PCR recorded on the national database and categorised as symptomatic or asymptomatic based on self-report. Severe infection was defined as an admission to hospital with ICD-10 code U07.1 on a date occurring between 1 day prior to the test and 2 weeks after. Respondents who reported having had a positive test that was not recorded on the database were excluded from the study as we could not determine whether they were incorrect or had taken the test outside Scotland.

Socioeconomic deprivation was obtained from postcode of residence using the Scottish Index of Multiple Deprivation derived from aggregated data on: income, employment, education, health, access to services, crime and housing^23^. SARS-CoV-2 variants were defined as dominant if they accounted for ≥95% of cases genotyped that week (https://sars2.cvr.gla.ac.uk/cog-uk/). Pre-existing health conditions were ascertained from self-report, previous hospitalizations and dispensed prescriptions. Respiratory disease was defined as International Classification of Diseases 10 (ICD10) codes J40-J47, J98.2 or J98.3, or bronchodilators, inhaled corticosteroids, cromoglycate, leukotriene or phosphodiesterase type-4 inhibitor (British National Formulary (BNF) 3.1–3.3), or self-report. Coronary heart disease was defined as ICD10 codes I11.0, I13.0, I13.2, I20-I25 (excluding I24.1), I50, T82.2, or Z95.5, or self-report. Depression was defined as ICD10 codes F30-F33, or anti-depressant, hypnotic or anxiolytic (BNF 4.1;4.3), or self-report. Diabetes was defined as ICD10 codes E10-E14, G590, G632, H280, H360, M142, N083, O240-O243 or self-report^24^. The total number of self-reported health conditions was categorised as 0, 1, 2–3 or ≥4.

### Outcomes

The outcomes measured were 26 symptoms (harmonised with the ISARIC questionnaire)^25^, limitations across 7 activities of daily living, health-related quality of life (using EQ-5D), hospitalization, admission to an intensive care unit (ICU), and all-cause mortality in the whole cohort, as well as self-reported recovery status (full, partial or none) in the symptomatic infection group. Hospitalization (as an outcome) was defined as admission to hospital on a date at least two weeks following the index test, to exclude admissions related to the acute infection. Delayed recovery was defined as participants with previous symptomatic infection who reported no or partial recovery at their first follow-up but an improvement at subsequent follow-up; either from no to partial or full recovery, or from partial to full recovery. Relapse was defined as participants with previous symptomatic infections who reported full or partial recovery at their first follow-up but a deterioration at subsequent follow-up; either full to partial or no recovery, or from partial to no recovery.

### Statistical analyses

Baseline characteristics and crude outcomes broken down by infection status (symptomatic, asymptomatic or never infected) were summarized using frequencies/percentages and medians/inter-quartile ranges for categorical and continuous variables and compared using chi-square tests and Mann–Whitney U tests, respectively. A correlation matrix of current symptoms was used to produce a heat map of symptom clustering at follow-up. Separate binary logistic regression models were run in the whole cohort to determine the association between infection status and the outcomes of individual symptoms, limitations in daily activities, hospitalization, ICU admission and death, using never infected as the referent group. Poisson regression models were run for the outcome of EQ-5D because it was a numeric variable and did not satisfy the assumptions required for linear regression. All models were run univariately, then adjusted incrementally for: socioeconomic factors (age, sex, ethnic group, deprivation) and stage of follow-up (6, 12 or 18 months); pre-existing health conditions (count, respiratory and coronary heart disease, depression, diabetes); vaccination status; and dominant SARS-CoV-2 variant. In the symptomatic infection group, the same models were run to determine the factors associated with these outcomes and recovery status. All analyses were performed using Stata v16.

### Reporting summary

Further information on research design is available in the Nature Research Reporting Summary linked to this article.

## Supplementary information

Supplementary information

Reporting Summary

## Data Availability

The datasets analysed during the current study are available in the National Services Scotland National Safe Haven, https://www.isdscotland.org/Products-and-Services/eDRIS/Use-of-the-National-Safe-Haven/. This protects the confidentiality of the data and ensures that Information Governance is robust. Applications to access health data in Scotland are submitted to the NHS Scotland Public Benefit and Privacy Panel for Health and Social Care. Information can be found at https://www.informationgovernance.scot.nhs.uk/pbpphsc/.
