# Author Correction: Outcomes among confirmed cases and a matched comparison group in the Long-COVID in Scotland study

**DOI:** 10.1038/s41467-022-34344-z

**Published:** 2022-11-01

**Authors:** Claire E. Hastie, David J. Lowe, Andrew McAuley, Andrew J. Winter, Nicholas L. Mills, Corri Black, Janet T. Scott, Catherine A. O’Donnell, David N. Blane, Susan Browne, Tracy R. Ibbotson, Jill P. Pell

**Affiliations:** 1 Institute of Health and Wellbeing, University of Glasgow, Glasgow, G12 8RZ UK; 2 Emergency Department, Queen Elizabeth University Hospital, Glasgow, G52 4TF UK; 3 Public Health Scotland, Meridian Court, Glasgow, G2 6QQ UK; 4 School of Health and Life Sciences, Glasgow Caledonian University, Glasgow, G4 0BA UK; 5 Sandyford Sexual Health Services, NHS Greater Glasgow and Clyde, Glasgow, G3 7NB UK; 6 BHF Centre for Cardiovascular Science, University of Edinburgh, Edinburgh, EH16 4SU UK; 7 Usher Institute, University of Edinburgh, Edinburgh, EH16 4UX UK; 8 Aberdeen Centre for Health Data Science, University of Aberdeen, Aberdeen, AB25 2ZD UK; 9 Public Health Directorate, NHS Grampian, Aberdeen, AB15 6RE UK; 10 MRC-University of Glasgow Centre for Virus Research, University of Glasgow, Glasgow, G61 1QH UK

**Keywords:** Epidemiology, Risk factors, SARS-CoV-2

Correction to: *Nature Communications* 10.1038/s41467-022-33415-5, published online 12 October 2022

The original version of this Article contained an error in the Discussion, which incorrectly read ‘Lack of recovery was associated with more severe infection, older age, female gender, black and South Asian ethnic groups, deprivation, pre-existing respiratory disease and multimorbidity but pre-infection vaccination was associated with reduced risk of some persistent symptoms.’. The correct version states ‘white ethnicity’ in place of ‘black and South Asian ethnic groups’. This has been corrected in both the PDF and HTML versions of the Article.

