# Supplementary material for: Outcomes among confirmed cases and a matched comparison group in the Long-COVID in Scotland study
Source: Nat Commun. 2022 Oct 12;13:5663. doi: 10.1038/s41467-022-33415-5 (PMC9556711; doi:10.1038/s41467-022-33415-5)
Supplement: Supplementary file 1 — Supplementary information [file 41467_2022_33415_MOESM1_ESM.pdf]

Outcomes among confirmed cases and a matched comparison group in the Long-COVID in Scotland Study

Supplementary Table 1. Overall recruitment rates by follow-up time

		Test result		
Follow-up period		Negative	Positive	Total
6 months	Invitations	466,890	158,425	625,315
	Responses	60,413	30,165	90,578
	Response rate (%)	13%	19%	14%
12 months	Invitations	180,684	61,728	242,412
	Responses	14,886	7,077	21,963
	Response rate (%)	8%	11%	9%
18 months	Invitations	6,382	2,243	8,625
	Responses	621	313	934
	Response rate (%)	10%	14%	11%

Supplementary Table 2. Recovery status in symptomatic participants with serial questionnaire data, by follow-up time

	Participated at 6 and 12 months N=3,744		Participated at 12 and 18 months N=197	
	Recovery status at 6 months	Recovery status at 12 months	Recovery status at 12 months	Recovery status at 18 months
Fully recovered	1,683 (45%)	1,736 (46%)	76 (39%)	75 (38%)
Not recovered	295 (8%)	303 (8%)	21 (11%)	21 (11%)
Partly recovered	1,766 (47%)	1,705 (46%)	100 (51%)	101 (51%)

Supplementary Table 3. Crude outcomes in symptomatic participants with serial questionnaire data, by follow-up time

	Participated at 6 and 12 months N=3,744			Participated at 12 and 18 months N=197		
	6 months	12 months	*p*-value*	12 months	18 months	*p*-value*
Symptoms in last week	N (%)	N (%)		N (%)	N (%)	
Tiredness	1,831 (49)	1,812 (48)	0.552	100 (51)	105 (53)	0.515
Headache	1,009 (27)	1,029 (27)	0.510	53 (27)	59 (30)	0.387
Muscle aches/weakness	1,074 (29)	1,057 (28)	0.553	62 (31)	74 (38)	0.052
Joint pain	861 (23)	875 (23)	0.599	63 (32)	64 (32)	0.884
Breathless	928 (25)	906 (24)	0.403	49 (25)	56 (28)	0.237
Anxious/depressed	836 (22)	816 (22)	0.455	48 (24)	51 (26)	0.631
Confused/difficulty concentrating	575 (15)	534 (14)	0.065	31 (16)	30 (15)	0.847
Sleep problems	988 (26)	1,038 (28)	0.085	58 (29)	70 (36)	0.070
Dry cough	399 (11)	566 (15)	<0.001	31 (16)	30 (15)	0.873
Cough with phlegm	360 (10)	551 (15)	<0.001	17 (9)	30 (15)	0.016
Change in taste	465 (12)	345 (9)	<0.001	17 (9)	14 (7)	0.491
Change in smell	533 (14)	391 (10)	<0.001	21 (11)	15 (8)	0.201
Problems hearing	231 (6)	253 (7)	0.214	17 (9)	19 (10)	0.617
Problems with eyesight	315 (8)	289 (8)	0.165	16 (8)	21 (11)	0.251
Pins and needles	477 (13)	460 (12)	0.439	36 (18)	33 (17)	0.578
Chest pain	274 (7)	284 (8)	0.565	≤10	19 (10)	-
Palpitations	348 (9)	377 (10)	0.123	32 (16)	32 (16)	1.000
Poor appetite	218 (6)	235 (6)	0.326	15 (8)	18 (9)	0.513
Abdominal pain	276 (7)	314 (8)	0.048	17 (9)	16 (8)	0.835
Sickness/vomiting	239 (6)	245 (7)	0.742	16 (8)	19 (10)	0.439
Diarrhea	323 (9)	352 (9)	0.160	27 (14)	20 (10)	0.162
Constipation	197 (5)	192 (5)	0.759	16 (8)	11 (6)	0.225
Weight loss	79 (2)	85 (2)	0.567	≤10	≤10	-
Dizzy/blackouts/fits	194 (5)	179 (5)	0.346	15 (8)	14 (7)	0.796
Balance problems	243 (6)	267 (7)	0.174	15 (8)	21 (11)	0.157

Skin rash	174 (5)	150 (4)	0.088	≤10	≤10	-
No symptoms now	996 (27)	1,008 (27)	0.669	52 (26)	54 (27)	0.763
Difficulties with activities of daily living						
Walking; getting around	809 (22)	807 (22)	0.932	45 (23)	54 (27)	0.106
Housework; DIY; chores	870 (23)	876 (23)	0.807	57 (29)	56 (28)	0.869
Working; studying	860 (23)	818 (22)	0.104	64 (32)	55 (28)	0.139
Washing; dressing	251 (7)	282 (8)	0.042	15 (8)	23 (12)	0.033
Exercise; sport	1,068 (29)	1,080 (29)	0.663	60 (30)	64 (32)	0.537
Hobbies	421 (11)	449 (12)	0.169	27 (14)	29 (15)	0.683
Relationships	429 (11)	479 (13)	0.014	25 (13)	31 (16)	0.239
EQ-5D	N=3,707	N=3,665		N=195	N=192	
Median (IQR)	75 (55-85)	73 (52-85)	<0.001	70 (50-80)	66 (50-80)	0.667

* Wilcoxon signed-rank test for EQ-5D; McNemar's χ^2 test for other variables. All tests are two-sided.

Supplementary Figure 1. Self-completed Questionnaire

Q1. Have you ever had a positive Covid-19 test? (yes/no)

Q2. Have you had a Covid-19 vaccine? (yes/no)

Q3. What Covid-19 symptoms did you have? [only if Q1 yes] (select all that apply)
Fever; Cough; Change in taste; Change in smell; Tiredness (fatigue); Headache; Ear pain; Runny nose; Muscle aches or weakness; Joint pain; Breathlessness; Chest pain; Sore throat; Hoarse voice; Loss of appetite; Stomach (abdominal) pain; Diarrhoea; Confusion; Seizures; Hair loss; Unconscious/semi-conscious; I didn't have any symptoms [go to Q5]

Q4. How long did your symptoms last?
Less than 1 week;1-4 weeks; More than 4 weeks

Q5. How do you feel now?
Fully recovered; Partly recovered; Not recovered

Q6. Before your Covid-19 test, did you have any health issues or medical conditions?
Yes; No [go to Q8]

Q7. Which health issues or medical conditions did you have? (select all that apply)
Arthritis; Asthma/bronchitis/COPD; Cancer; Cystic fibrosis; Deep vein thrombosis; Depression/anxiety; Diabetes; Heart disease; Heart failure; High blood pressure; HIV; Home oxygen; Kidney disease; Liver disease; Neurological condition; Overweight; Obese; Pulmonary embolism; Pulmonary fibrosis; Stroke; I didn't have any of these [go to Q8]

Q8. Have you experienced any of the following in the last week? (select all that apply)
Dry cough; Cough with phlegm; Change in taste; Change in smell; Problems hearing; Problems with eyesight; Tired; Headache; Muscle aches/weakness; Pins and needles; Joint pain; Breathless; Chest pain; Palpitations; Poor appetite; Stomach (abdominal) pain; Feeling sick/vomiting; Diarrhoea; Constipation; Weight loss; Anxious/depressed; Confusion/difficulty concentrating; Dizzy/blackouts/fits; Balance problems; Skin rash; Problems sleeping; I haven't experienced any of these [go to Q10]

Q9. Are any of these new or worse since your Covid-19 test? (select all that apply)
Response list from Q8; None of these are new or worse [go to Q10]

Q10. Is your current health causing you difficulty with any of the following? Select all that apply.
Walking/getting around; Housework/DIY/chores; Working/studying; Washing/dressing; Exercise/sports; Hobbies; Relationships; None of these apply [go to Q12; if also no to Q2 go to Q13]

Q11. Which of the difficulties you're experiencing are new/worse since having your Covid-19 test? (select all that apply)
Response list from Q10; None of these are new or worse [go to Q13]

Q12. Has your job changed since your Covid-19 test?

No change; Different due to my Covid-19 illness; Different for other reasons; None of these apply

Q13. What is your ethnicity?
White Scottish; White Other British; White Irish; White Gypsy/Traveller; White Polish; Other white ethnic group; Mixed or multiple ethnic groups; Pakistani, Pakistani Scottish or Pakistani British; Indian, Indian Scottish or Indian British; Bangladeshi, Bangladeshi Scottish or Bangladeshi British; Chinese, Chinese Scottish or Chinese British; Other Asian, Asian Scottish or Asian British; African, African Scottish or African British; Other African; Caribbean, Caribbean Scottish or Caribbean British; Black, Black Scottish or Black British; Other Caribbean or Black; Arab, Arab Scottish or Arab British; Other ethnic group; Prefer not to say

Q14. How would you rate your health today?
EQ-5D thermometer (0 Worst health I can imagine – 100 Best health I can imagine)

Supplementary Figure 2. Heat map of current symptom clusters among people who had symptomatic COVID-19 infection

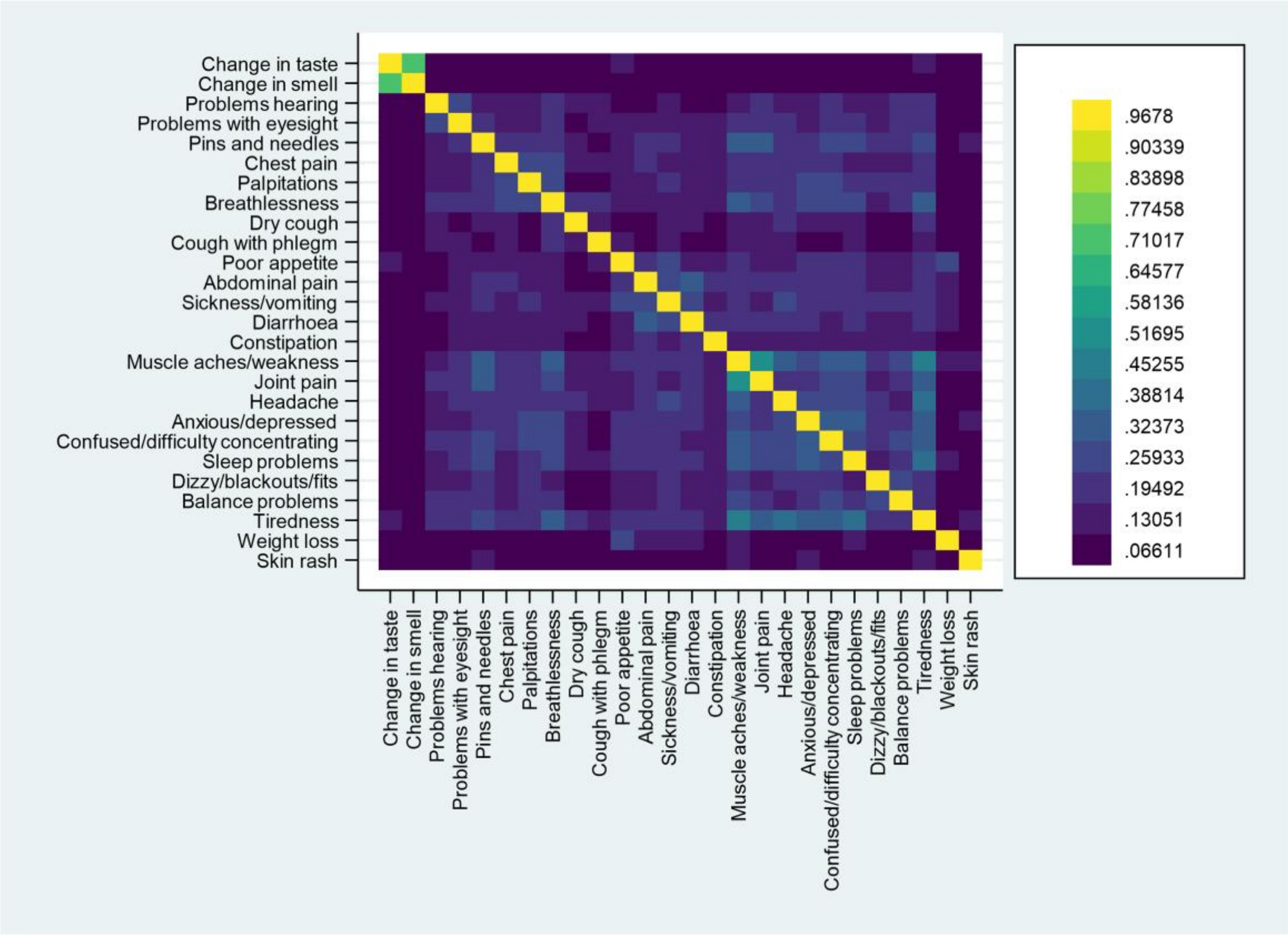

Values refer to the correlation coefficient.
