# Supplementary material for: Outcomes among confirmed cases and a matched comparison group in the Long-COVID in Scotland study
Source: Nat Commun. 2022 Oct 12;13:5663. doi: 10.1038/s41467-022-33415-5 (PMC9556711; doi:10.1038/s41467-022-33415-5)
Supplement: Supplementary file 2 — Reporting Summary [file 41467_2022_33415_MOESM2_ESM.pdf]

nature research

Corresponding author(s): Jill P Pell

Last updated by author(s): Sep 5, 2022

Reporting Summary

Nature Research wishes to improve the reproducibility of the work that we publish. This form provides structure for consistency and transparency in reporting. For further information on Nature Research policies, see our Editorial Policies and the Editorial Policy Checklist.

Statistics

For all statistical analyses, confirm that the following items are present in the figure legend, table legend, main text, or Methods section.

n/a	Confirmed	
☐	☒	The exact sample size (n) for each experimental group/condition, given as a discrete number and unit of measurement
☒	☐	A statement on whether measurements were taken from distinct samples or whether the same sample was measured repeatedly
☐	☒	The statistical test(s) used AND whether they are one- or two-sided<br>*Only common tests should be described solely by name; describe more complex techniques in the Methods section.*
☐	☒	A description of all covariates tested
☐	☒	A description of any assumptions or corrections, such as tests of normality and adjustment for multiple comparisons
☐	☒	A full description of the statistical parameters including central tendency (e.g. means) or other basic estimates (e.g. regression coefficient) AND variation (e.g. standard deviation) or associated estimates of uncertainty (e.g. confidence intervals)
☐	☒	For null hypothesis testing, the test statistic (e.g. F, t, r) with confidence intervals, effect sizes, degrees of freedom and P value noted<br>*Give P values as exact values whenever suitable.*
☒	☐	For Bayesian analysis, information on the choice of priors and Markov chain Monte Carlo settings
☒	☐	For hierarchical and complex designs, identification of the appropriate level for tests and full reporting of outcomes
☒	☐	Estimates of effect sizes (e.g. Cohen's d, Pearson's r), indicating how they were calculated

Our web collection on statistics for biologists contains articles on many of the points above.

Software and code

Policy information about availability of computer code

Data collection	No software used.
Data analysis	Stata version 16.0

For manuscripts utilizing custom algorithms or software that are central to the research but not yet described in published literature, software must be made available to editors and reviewers. We strongly encourage code deposition in a community repository (e.g. GitHub). See the Nature Research guidelines for submitting code & software for further information.

Data

Policy information about availability of data

All manuscripts must include a data availability statement. This statement should provide the following information, where applicable:

- Accession codes, unique identifiers, or web links for publicly available datasets
- A list of figures that have associated raw data
- A description of any restrictions on data availability

The datasets analysed during the current study are available in the National Services Scotland National Safe Haven, https://www.isdscotland.org/Products-and-Services/eDRIS/Use-of-the-National-Safe-Haven/.

Field-specific reporting

Please select the one below that is the best fit for your research. If you are not sure, read the appropriate sections before making your selection.

☐ Life sciences ☒ Behavioural & social sciences ☐ Ecological, evolutionary & environmental sciences

For a reference copy of the document with all sections, see nature.com/documents/nr-reporting-summary-flat.pdf

Behavioural & social sciences study design

All studies must disclose on these points even when the disclosure is negative.

Study description	Nationwide ambidirectional cohort study (quantitative data).
Research sample	Every adult (>16 years) in Scotland with a positive PCR test for SARS-CoV-2 from April 2020 to May 2021 was invited to participate along with a comparison group who had had a negative test but never had a positive test, matched 3:1 by age, sex, and area-based socioeconomic deprivation quintile. Overall response rate was 16%. The study cohort comprised 96,238 participants. Their median age at baseline was 45 (IQR 31-56) years, 39% were male, 91% white. Compared with those who did not provide consent, responders were more likely to be female (60.9% vs 51.2%; p-value <0.001), were older (>40 years 59.5% vs 46.0%; p-value <0.001) and less deprived (most deprived SIMD quintile 24.0% vs 27.0%; p-value <0.001).
Sampling strategy	There was no predetermined sample size. This is a nationwide study that invited every adult in Scotland who had a positive PCR test for SARS-CoV-2 from April 2020 to May 2021 along with a comparison group. There is no sampling procedure. Everyone in the population with a positive Covid-19 test was invited.
Data collection	A self-completed online questionnaire collected information on pre-existing health conditions at the time of the index test (first positive test or, for comparison group, most recent negative test) as well as current symptoms, limitations in daily activities and quality of life. Those who had tested positive also provided information on symptoms during the initial infection and current recovery status. Questionnaires were completed 6, 12, and 18 months after the index test. Data is collected via an online questionnaire. The researcher is not present with the participant. There is no experimental condition. Additional data were obtained through linkage to electronic health records both five years prior their index test and subsequent to the test (up to January 2022) on hospitalizations (Scottish Morbidity Record 01/04), dispensed prescriptions (Prescribing Information System), vaccinations, and death certificates (General Registrar Office).
Timing	Questionnaire responses were collected between May and October 2021.
Data exclusions	We excluded 6,235 participants who had only negative tests recorded but self-reported they had tested positive. This exclusion criterion was established in advance of data collection.
Non-participation	Response rate 16%.
Randomization	This is an observational study with group membership determined by Covid test result. Participants were not allocated into experimental groups. Covariates were controlled either by matching (sex, age, and socioeconomic deprivation), or model adjustment for all other potential confounders.

Reporting for specific materials, systems and methods

We require information from authors about some types of materials, experimental systems and methods used in many studies. Here, indicate whether each material, system or method listed is relevant to your study. If you are not sure if a list item applies to your research, read the appropriate section before selecting a response.

Materials & experimental systems

n/a	Involved in the study	
☒	☐	Antibodies
☒	☐	Eukaryotic cell lines
☒	☐	Palaeontology and archaeology
☒	☐	Animals and other organisms
☐	☒	Human research participants
☒	☐	Clinical data
☒	☐	Dual use research of concern

Methods

n/a	Involved in the study	
☒	☐	ChIP-seq
☒	☐	Flow cytometry
☒	☐	MRI-based neuroimaging

Human research participants

Policy information about studies involving human research participants

Population characteristics	See above.
Recruitment	Potential participants were sent invitations to participate by SMS message. Participation is voluntary, therefore there is self-selection bias. Furthermore, as detailed above responders differ from the general population in some ways.
Ethics oversight	Study approval was obtained from the West of Scotland Research Ethics Committee (ref.170 21/WS/0020) and the Public Benefit and Privacy Panel (ref. 2021-0180).

Note that full information on the approval of the study protocol must also be provided in the manuscript.
